# Subcellular nanoparticle trafficking investigated with label-free, live cell imaging

**DOI:** 10.1039/d5nh00749f

**Published:** 2026-04-07

**Authors:** Elizabeth B. Nelson, Gil Covarrubias, Namita Nabar, Victoria Gomerdinger, Anderson Scott, Paula T. Hammond, Joelle P. Straehla

**Affiliations:** 1. Koch Institute for Integrative Cancer Research, Massachusetts Institute of Technology, Cambridge, MA 02139, USA.; 2. Department of Chemical Engineering, Massachusetts Institute of Technology, Cambridge, MA 02139, USA.; 3. Ben Towne Center for Childhood Cancer Research, Seattle Children’s Research Institute, Seattle, WA 98105, USA.; 4. Department of Pediatrics, Seattle Children’s Hospital, University of Washington, Seattle, WA 98105, USA.; 5. Broad Institute of MIT and Harvard, Cambridge, MA 02139, USA.; 6. Institute for Soldier Nanotechnologies, Cambridge, MA 02139, USA.

**Keywords:** nanoparticle imaging, holography, tomography, label-free, refractive index

## Abstract

Nanoparticle drug delivery systems have significant potential to transform precision medicine due to their ability to encapsulate a wide range of cargo, improve systemic circulation time, and enhance targeted delivery. These delivery properties can be further modified by altering the surface chemistry of the nanoparticle carrier. However, a gap remains in our understanding of the cellular mechanisms underlying nanoparticle uptake, in addition to the subcellular trafficking kinetics. A comprehensive understanding of nanoparticle-cell interactions considering both the nanocarrier and the drug cargo has been challenging, in part due to technological limitations. Here, we present a robust imaging workflow to study long-term dynamics of nanoparticle delivery in live cells. We show that integration of holography and tomography enhances the study of live cells in a label-free environment and can be combined with intermittent fluorescence microscopy to assess nanoparticle uptake and delivery kinetics for up to 30 hours. We also describe a method to quantitatively characterize uptake of a library of fluorescently tagged lipid- and polymer-based nanoformulations without introducing cell or organelle markers. This application of dynamic fluorescent holotomography as a novel method to investigate nanoparticle uptake and cargo delivery highlights the expanding utility of multimodal, label-free, live imaging techniques.

## Introduction

Designed to encapsulate a range of therapeutic cargos, nanoparticle (NP) delivery platforms have the potential to transform precision medicine ^[1,2]^. NPs represent a diverse class of therapeutics, conventionally defined by size up to 100 nanometers (nm) and categorized by chemical composition. Many NPs in clinical use can be categorized as lipid-based, polymeric, or inorganic/metallic ^[3]^. Lipid-based NPs can be further classified by structure, such as micelles, liposomes, or solid lipid NPs (LNPs), and have proven to be efficient delivery carriers for small molecules and nucleic acids ^[4]^. The United States Food and Drug Administration (FDA) and the European Medicines Agency (EMA) have approved several lipid-based nanoformulations for clinical use, including mRNA LNP vaccines against COVID-19 and small molecule-loaded liposomes used to treat a variety of cancers ^[5,6]^. The diverse potential cargo and promising biocompatibility of lipid-based NPs make them a compelling platform to address a variety of diseases.

The material properties of a given NP formulation and its surface chemistry influence cellular interactions and uptake ^[7]^. Within classes of nanoscale carriers, there is a range of design parameters that can be tuned for targeted delivery. Current preclinical research efforts screen large libraries of NP formulations to identify NPs that enhance cell and tissue targeting, improve efficiency of cargo delivery, and reduce off-target toxicity ^[8,29,35]^. Clinically available lipid-based NPs consist of a narrow repertoire of lipids and often include the stabilizing surface polymer poly(ethylene glycol) (PEG), known to prolong circulation time by shielding against cellular interactions ^[9]^. Other physicochemical properties, such as stiffness, play a role in cellular internalization and biodistribution with differential interactions across cell and tissue types ^[10,11]^. Chemically modifying the outer surface of the particle also influences both uptake and intracellular trafficking with the potential to improve specificity for different cell types ^[12,30]^. Exploring the influence of NP core composition and outermost surface chemistry on cellular uptake using high-throughput methods has shed light on the analogous role of biological factors underlying cellular delivery ^[13,14]^, but these data remain limited by poor temporal resolution. Long-term live cell imaging is a promising technology that can be leveraged to further our understanding of cell-NP interactions.

Live cell imaging is a tool of growing interest in characterizing uptake mechanisms and temporal dynamics of NP formulations. Particularly for lipid-based NPs, fluorescence microscopy is a commonly used methodology for studying NP uptake mechanisms and trafficking in cells ^[15]^. Modalities like confocal microscopy or fluorescence recovery after photobleaching (FRAP) require sample-dependent optimization and a powerful illumination source prone to cell phototoxicity ^[16,17]^. These techniques typically require fixation or chemical staining, limiting the ability to study NP uptake and cargo delivery over time. Additionally, the chemicals required to fix or permeabilize cells can interfere with the composition of lipid-based NPs ^[18]^. Recent advances using label-free imaging overcome these limitations and reveal new insights into uptake, intracellular trafficking, and delivery of various NP formulations in live cells ^[31,32]^. Specifically, advanced quantitative fluorescence microscopy tools are evolving to enable high spatial and temporal resolution of interactions between living cells and nanotherapeutics. Novel instrumentation combining holography (interference phenomena) and tomography (3D reconstruction from 2D slices) enables long-term, live-cell imaging using intrinsic optical properties, allowing label-free observation of cells *in vitro*. In this communication we combine 3D holotomography (HT) with intermittent fluorescence to investigate NP uptake and therapeutic delivery in live, unlabeled cancer cells over prolonged time periods. We use dynamic fluorescence HT to investigate cellular dynamics, uptake of NPs, dose-response kinetics of therapeutic NP formulations, and NP-mediated delivery of nucleic acid cargo in live cells. In an important step toward high-throughput screening, we describe a semi-automated workflow for the acquisition and analysis of cell-NP interactions, enabling quantitative analysis of uptake, cell death, and cargo delivery over time. This technology complements existing methods for studying cell-cell and cell-NP interactions and has the potential to enhance NP characterization for high-throughput screening or mechanistic studies.

## Materials and methods

### CELL CULTURE:

Well-characterized cancer cell lines were chosen for reproducibility. Breast cancer (T47D, #HTB-133) and neuroblastoma (SKN-BE2, #CRL-2271) cells were sourced from ATCC. Melanoma cells were sourced from Sigma-Aldrich (LOX-IMVI, #SCC201). Cells overexpressing the solute carrier SLC46A3 were generated using lentiviral transduction, as described in prior work^[13]^. Cells were cultured at 37°C and 5% CO_2_ in RPMI-1640 media (ATCC, #30–2001) with 10% Fetal bovine serum (FBS, Seradigm, #1500–500) and 1% penicillin/streptomycin (Corning, #30–002-Cl). Each cell line was verified mycoplasma negative and STR-validated. Cells were maintained in culture and seeded 24–48 hours prior to the time of acquisition. Cells were seeded into 35mm glass-bottom dishes (Ibidi, #80136) in 800 to 1000 μL of complete media at a density between 5E+4 to 1E+5 cells, depending on the assay parameters, such as duration or viability studies. Media was carefully changed immediately before image acquisition to remove dead cells or debris. For assays involving NP uptake or chemotherapeutic treatment, the desired concentration was diluted into the media during this step to be dosed in real time (deemed t=0).

### NANOPARTICLES AND CHEMOTHERAPEUTICS:

NPs described in Table S1 were diluted with DI water and characterized using dynamic light scattering (DLS, Malvern Zetasizer Advanced Pro, λ = 633 nm). Bare liposomes were synthesized using a thin-film hydration technique as described previously ^[13]^. Carboxylated polystyrene particles were purchased from a commercial vendor and used without modification (Invitrogen, #F8803). Solid LNPs were synthesized with 50 mol% ALC-0315 (Avanti, #890900), 38.5 mol% cholesterol (Avanti, #700000), 9.5 mol% DOPE (Avanti, #850725), 0.5 mol% Cy5-tagged DOPE (Avanti, #810335), and 1.5 mol% DMG-PEG 2000 (Cayman Chemical Co, #33945) as described previously ^[33]^. LNPs were prepared with 40 ng/μL mRNA encoding green fluorescent protein (GFP; TriLink Biotech, #L-7201-100) and dosed at 100 ng RNA per well. Layer-by-layer (LbL) doxorubicin-loaded liposomes were synthesized via self-assembly of 1 mol% DSPE-mPEG (Avanti, #880128), 5 mol% POPG (Avanti, # 840457), 54 mol% DPPC (Avanti, # 850355), and 40 mol% % cholesterol (Avanti, #700000) in ammonium sulfate, then extruded through a 100nm membrane. Dialysis was used to remove ammonium sulfate external to the liposome core before remote loading of doxorubicin at 65°C overnight. After further dialysis to remove unencapsulated doxorubicin, liposomes were layered with polymers using LbL assembly with microfluidic chips as previously described ^[34]^. First, poly-L-arginine (PLR) was adsorbed, followed by either poly-L-glutamate (PLE) or hyaluronic acid (HA). Charge conversion, particle size, and polydispersity index were assessed with Malvern Zetasizer Advanced Pro DLS (Table S1). Drug concentrations for the synthesized LbL doxorubicin-liposomes were calculated, following methanol digestion, based on a doxorubicin fluorescent standard curve. Commercial liposomal doxorubicin (Doxil) was purchased as a clinical-grade compound from Baxter. CPX-351 clinical grade compound (Vyxeos) was provided through a materials transfer agreement with Jazz Pharmaceuticals.

### MICROSCOPY:

Cells were imaged in a humidified, temperature-controlled chamber incubator (Tokai Hit) maintained at 37°C with 5% CO_2_ supply. Temperature stabilization for 10–30 minutes is necessary to avoid fog interference before acquiring images. All images and movies were acquired on the 3D Cell Explorer 96focus microscope (Nanolive SA, Switzerland). Acquisitions using fluorescence were captured with filters for FITC or Cy5. The focus in the RI channel is user-defined at several points of reference per condition before the acquisition. The fluorescence plane is then defined based on the z-offset relative to the RI channel. Further detailed acquisition parameters and experimental conditions can be found in Table S2.

### IMAGE ANALYSIS AND FIJI PROCESSING:

Grid scan stitching and fluorescence channel registration were performed automatically using Nanolive SA proprietary analysis software (EVE). Automated analysis programs (LIVE Cytotoxicity Assay, LIVE Cell Death Assay, or the SMART Lipid Droplet Assay ^LIVE^ (all Nanolive SA)) were applied to the raw imaging data for automated segmentation and quantification of cells and lipid droplets, and for automated classification of cells into living, apoptotic, or necrotic states. The software utilized is based on a proprietary deep learning algorithm that was trained with an extensive dataset (> 3000 TB, Nanolive SA). Further details are available from the vendor, as described in the Nanolive Technical Note for the LIVE Cytotoxicity Assay. All movies with fluorescent channels were processed in FIJI using ‘Combine Stacks’. For visualization after analysis, fluorescence and brightfield refractive index images were auto-adjusted for brightness and contrast.

## Results and Discussion

### Long-term, label-free visualization of cellular features

Label-free, live cell imaging has emerged as an important tool in cell biology within the last two decades, advancing the understanding of cell motility, mitosis, and response to external stimuli ^[19,20]^. Observing NPs in this context is of great interest but has largely been restricted to metallic NPs with innate properties compatible with label-free imaging ^[21,22,23]^. Visualization is also limited by conventional fluorescence imaging modalities that can either require sample fixation or short-term acquisitions to mitigate photobleaching.

**Figure 1.**
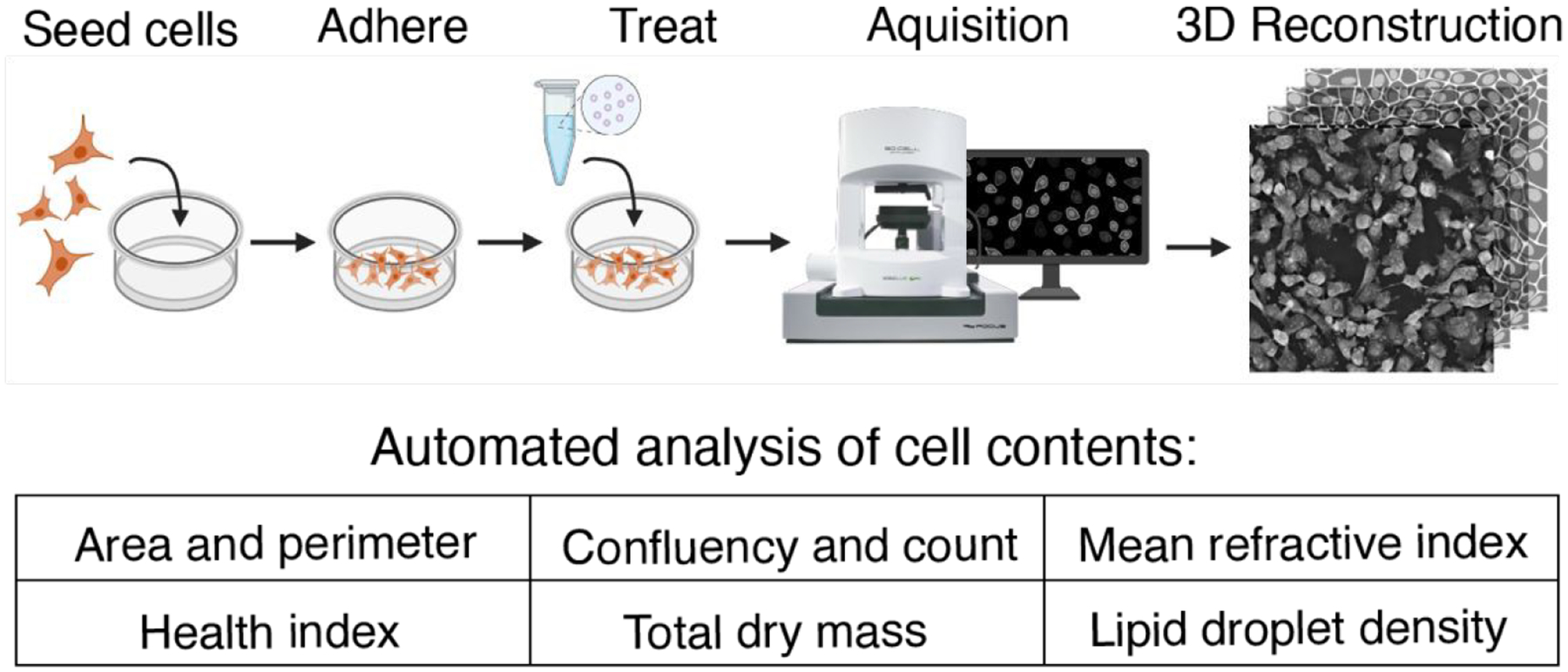
Schematic of the experimental design, beginning with cell culture in glass-bottom dishes, then image acquisition under controlled temperature and CO_2_, and concluding with post-processing using an automated analysis software to quantify cellular content and calculated metrics.

Holotomography is a newer approach that provides an image map of refractive index differences within a sample. Using a holotomographic microscope that combines three-dimensional (3D) HT with intermittent fluorescence microscopy, we performed long-term, label-free imaging of cellular dynamics and concurrently assessed the delivery of lipid-based NP formulations for up to 30 hours. The low-power nature of the illumination source reduces the effects of phototoxicity, allowing for longitudinal observation of morphological characteristics, lipid contents, or viability studies (Figure 1).

**Figure 2.**
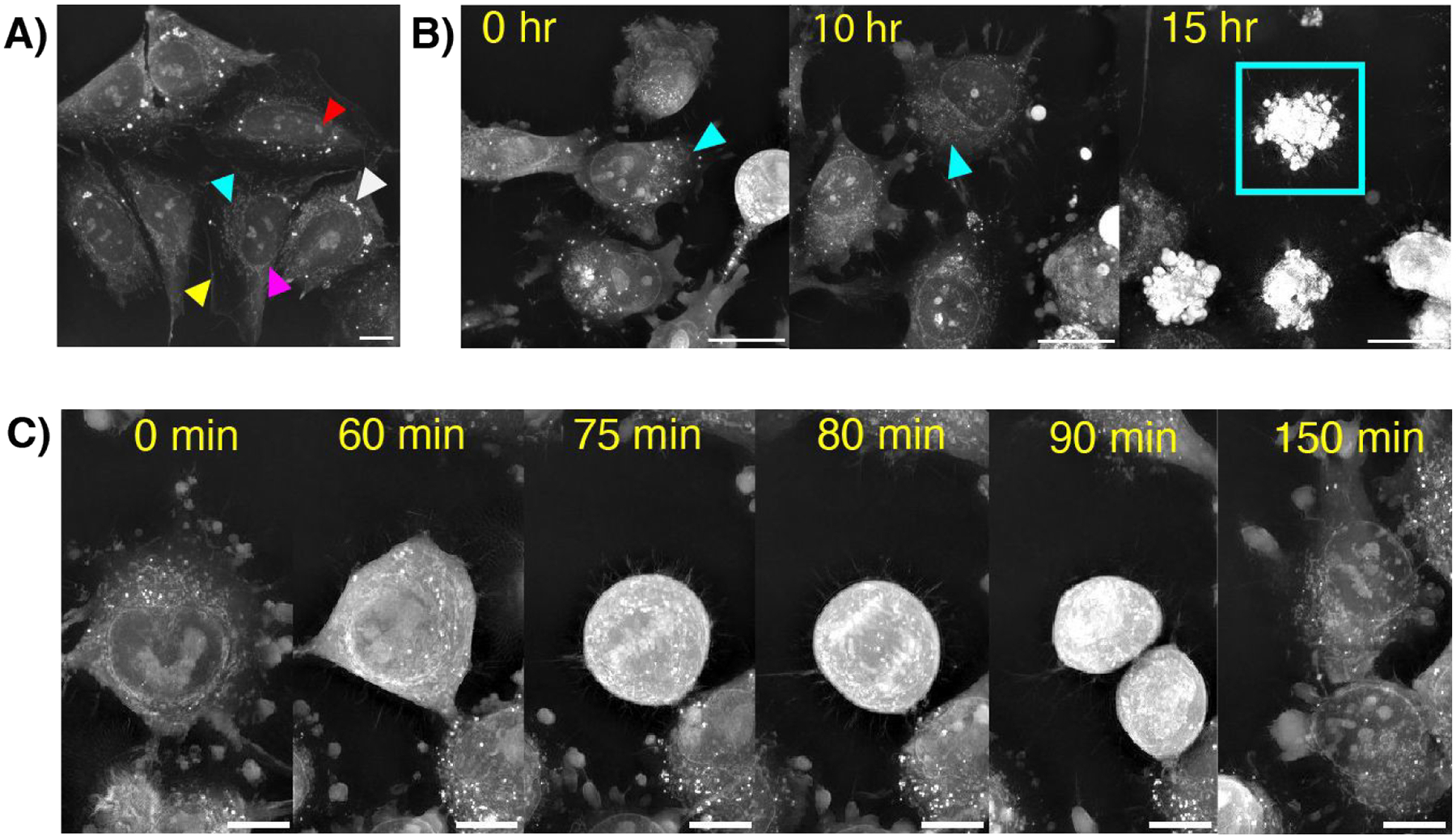
A) Unlabeled, untreated SKN-BE2 cells in the refractive index channel. Organelles highlighted by arrows include the cell membrane (yellow), lipid droplets (white), nucleus (magenta), nucleolus (red), and mitochondrial network (cyan); Movie (6 frames per second) 10 μm scale bar. B) LOX cells after treatment with 10 μM of doxorubicin at 0, 10, and 15 hours. Cyan arrows indicate mitochondrial networks. An apoptotic cell is outlined in the cyan square; 20 μm scale bar. C) Untreated LOX cell undergoing mitosis; 10 μm scale bar. Movie (6 fps, 10 μm scale bar). Movie (6 fps, 20 μm scale bar).

While the use of fluorescence is necessary to observe NP signal, holotomography can be used to assess changes in cell morphology and subcellular contents after dosing with NPs from either a single-cell region of interest (ROI) to a population-level field of view (FOV). For this technique, refractive index (RI) properties of intracellular features are leveraged in contrast against a reference signal, allowing for the visualization and quantification of subcellular organelles down to single lipid droplets ^[24,25,26]^. Computational analysis software (Nanolive’s digital assays) enables automated, consistent evaluation of quantifiable cell and organelle metrics. Without fixation or staining, we readily identified cellular features in cultured cancer cell lines, including the cell membrane, nucleus and nucleolus, lipid droplets, and mitochondrial networks in an established neuroblastoma cell line (Figure 2A). We next assessed the impact of cytotoxic drugs on cell morphology. In T47D breast cancer and LOX-IMVI melanoma cell lines, we observed characteristic disruption of mitochondrial networks and increased membrane blebbing following treatment with doxorubicin (Figure 2B) Under control conditions, we captured the phases of mitosis with high spatial and temporal resolution, clearly visualizing nuclear condensation in prophase and chromosomal segmentation in anaphase (Figure 2C).

### Cellular interaction with fluorescent nanoparticles

**Figure 3.**
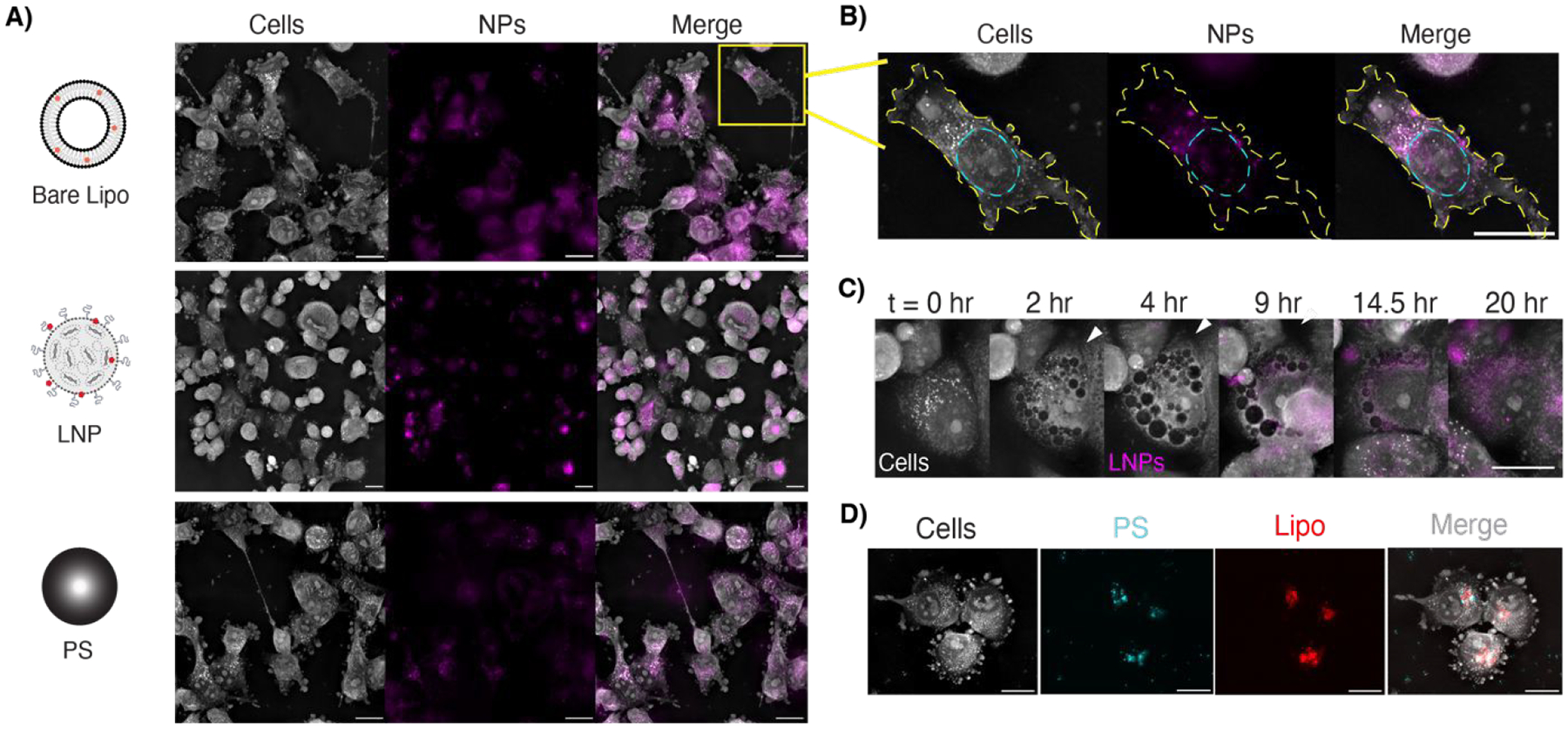
A) Representative images of LOX cells treated with equivalent concentrations of fluorescently labeled liposomes, LNPs, or PS NPs. Fluorescence channel is pseudo-colored in magenta. All scale bars are 20 μm. B) Individual cell with the membrane traced in yellow and the nucleus traced in cyan, showing liposomal distribution throughout the cytoplasm. C) LNP uptake in T47D cells via macropinocytosis (white arrow) over a 20-hour period. Movie (8 fps, 20 μm). D) LOX cells co-treated with PS NPs (labeled for FITC, shown in cyan) and Lipo NPs (labeled with Cy5, shown in red) with colocalization appearing as white in the merged image.

For optimal imaging conditions with HT, cells should be seeded in a monolayer without overcrowding by the end of the experimental imaging duration. We thus optimized seeding densities for each cell line for short- and long-term imaging to achieve a 70–85% confluent monolayer at the time of imaging. To investigate dynamic cellular interactions with NPs, we synthesized two fluorescent lipid-based formulations, liposomes and LNPs, and compared with carboxylated polystyrene (Figure 3A). We combined intermittent fluorescence imaging with holotomography by carefully tuning laser exposure settings to avoid phototoxicity and observed cells over 20 hours (Figure S1). Optimal imaging parameters were identified for each fluorescent NP; capturing fluorescent images every 7–10 minutes and maintaining the laser power below 15% enabled long-term imaging without impacting cell viability due to phototoxicity (Table S2).

Using this technique, we overlay NP location onto high-resolution HT images to observe puncta of fluorescence signal. For the liposomal NP formulation, we found that NPs partitioned into the cytoplasm roughly 18 hours after dosing (Figure 3B). Intermittent fluorescence imaging also enabled us to investigate uptake mechanisms over time. Over 20 hours after dosing with LNPs, we observe membrane ruffling and the formation of NP-filled vesicles, which is suggestive of endocytic uptake (Figure 3C). Similar, though more sparse vesicles, are observed when cells are treated with bare liposomes (Figure S5). By using multiple fluorescent channels, we can track different NP components simultaneously. In one experiment, polystyrene NPs and liposomes colocalize within the cytosol in conglomerates suggestive of vesicles (Figure 3D). This ability to perform multiplexed imaging offers a powerful way to probe NP delivery, distribution, and heterogeneity, with potential for further studies to investigate subcellular trafficking of different NP cargos.

While dynamic fluorescent HT offers unique advantages over orthogonal tools for studying cell-NP interactions, it also has distinct limitations (Table 1). Both flow cytometry and HT with intermittent fluorescence enable quantitative analysis of cellular fluorescence. However, flow cytometry is limited to a single time point and cannot distinguish between NPs located on the cell surface as opposed to intracellularly. The primary challenges of current HT technology are limited sensitivity of visualizing individual NPs (typically 100–200 nm) using fluorescence microscopy at the diffraction limit (~250 nm) ^[27]^ while simultaneously imaging live cells over an extended period. We found a clear tradeoff between the detection of nanoparticles at early time points and the risk of phototoxicity, as their fluorescent signal was initially weak but became stronger over time as the nanoparticles concentrated into intracellular vesicles. Furthermore, because fluorescence is captured in a single plane, we are limited in our ability to capture NPs spatially distributed throughout the z-plane to overlap with the RI z-stack acquisitions. To improve resolution for endocytosis studies, shorter imaging duration or NPs that can be detected label-free, such as high refractive index NPs, could be considered.

### Cellular response to therapeutic nanoparticles

**Figure 4.**
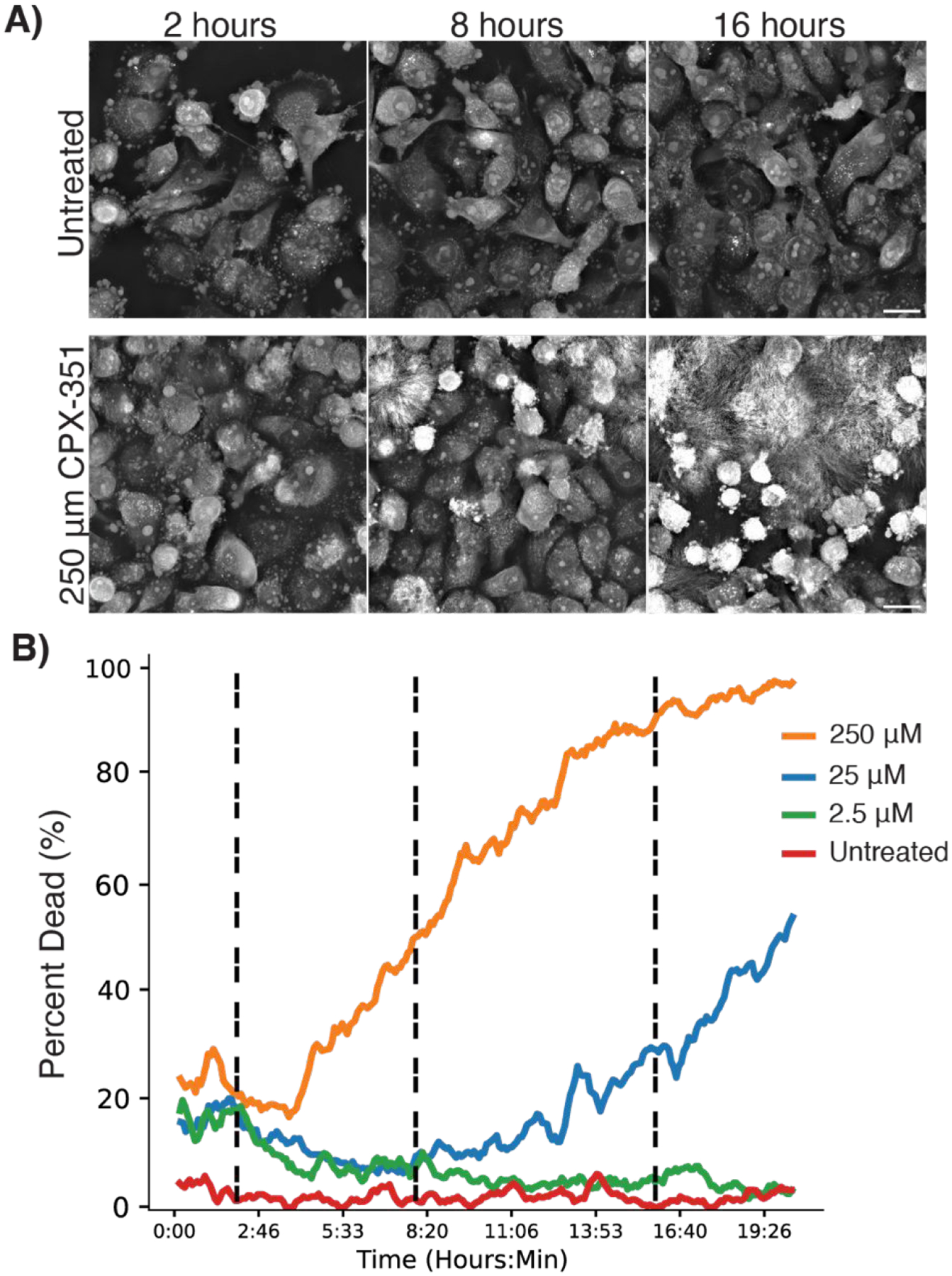
A) Representative images of LOX cells after 2, 8, and 16 hours when treated with CPX-351 (liposomal daunorubicin and cytarabine). B) Percent of dead cells evaluated with auto-segmentation over 20 hours when treated with increasing concentrations of CPX-351. Movies of the untreated and 250 μM groups (10 fps, time stamp in hours: minutes). All scale bars are 20 μm.

Having established the utility of dynamic HT for tracking fluorescently tagged NPs, we next shifted our focus to evaluating the impact of therapeutic NPs on cell morphology without fluorescence, thus eliminating the possibility of phototoxicity influencing the results. We used this approach to investigate the kinetics of apoptosis in cells treated with CPX-351, a chemotherapeutic NP formulation of daunorubicin and cytarabine. High doses of CPX-351 induced prominent changes consistent with apoptosis, including cell shrinking, membrane blebbing, and autofluorescence (Figure 4A). Automated cell segmentation and cell health indices were generated using the LIVE Cytotoxicity Assay software (Nanolive SA), confirming the expected dose-dependent kinetics of cell death following NP treatment with cytotoxic cargo (Figure 4B).

**Figure 5.**
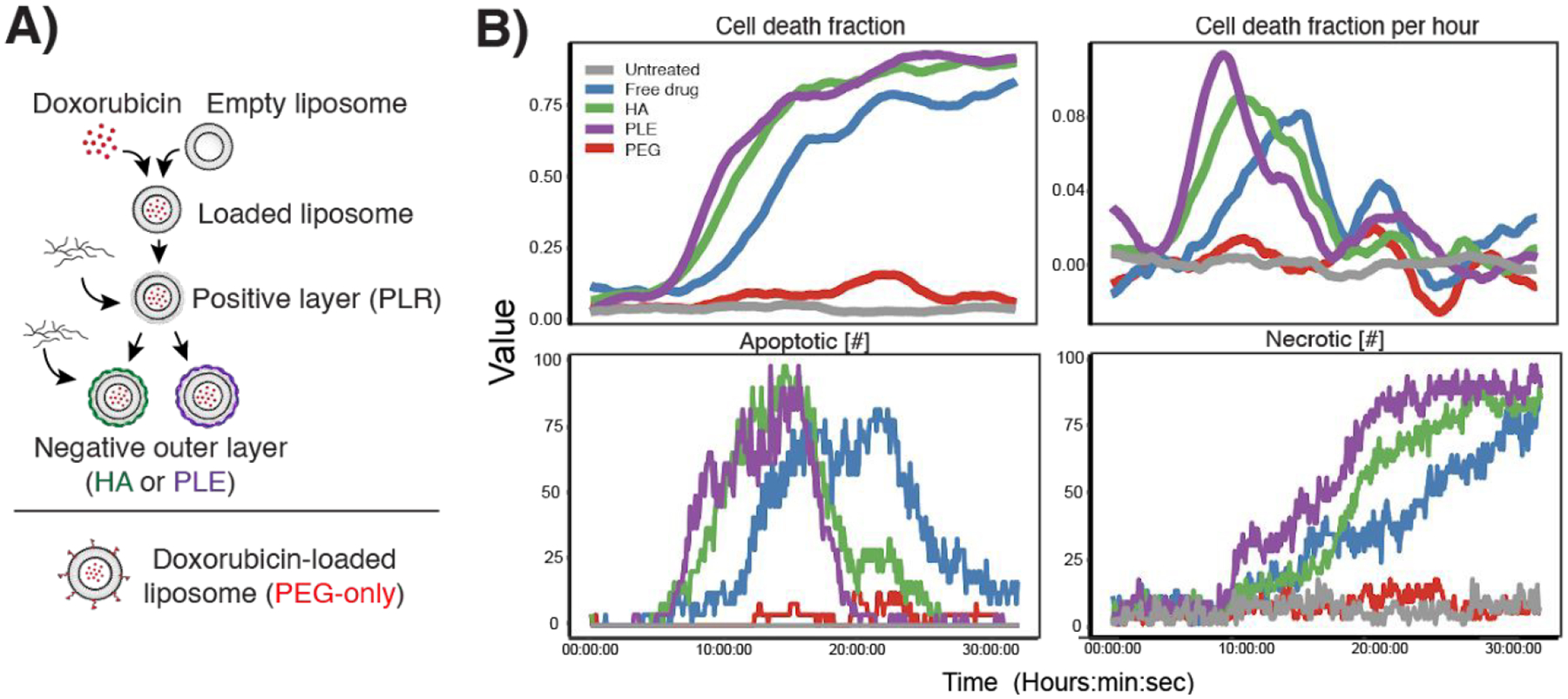
A) Schematic of doxorubicin-loaded liposome synthesis and layer-by-layer assembly and comparison to commercial PEG-liposomes. B) Plots representing cell death fraction, fraction per hour, percent of dead cells, and number of living cells over a 30-hour period when LOX cells were treated with the NPs from (A) at equivalent concentrations of doxorubicin. Movie of a sample well from the HA LbL group over 25 hours (8 fps, 20 μm scale bar).

We next generated a library of NPs containing the cytotoxic cargo doxorubicin and coated with different polymers to observe the impact of NP surface chemistry on cell death kinetics. We used layer-by-layer assembly to coat the surface of doxorubicin-loaded liposomes with nanometer-thick layers of charged polymers through alternating electrostatic adsorption. We chose hyaluronic acid (HA) and poly(L-glutamic acid) (PLE) as terminal outer layers based on previous work showing that these polymers modify NP uptake in a range of cancer cells ^[3,28,36]^. This assembly process enables direct comparison of each formulation with identical NP cores, differing only in outer layer surface chemistry. We included a commercial doxorubicin-loaded liposome with polyethylene glycol (PEG) surface coating and free drug at an equimolar drug concentration as controls (Figure 5A). Because we have previously shown that PLE and HA modulate nanoparticle-cell association, we hypothesize that these formulations would also modulate the kinetics of delivery and cell death. Using this established library to deliver a therapeutic cargo allows us to evaluate the utility of this automated workflow. Employing the same label-free imaging methodology as described above, we observed clear differences in LOX cellular response to each formulation over a 30-hour imaging period. The PEGylated liposomal doxorubicin did not lead to cell death during this time period and was similar to an untreated control, whereas free doxorubicin and polymer-coated doxorubicin liposomes were all cytotoxic (Figure 5B). Automated image analysis identified rapid onset of action of HA- and PLE-coated NPs as evidenced by an earlier peak in the cell death fraction per hour compared to free doxorubicin. Cells exhibited morphology consistent with apoptotic and necrotic cell death during the experiment (Figure 5A–B).

This label-free assay highlights key principles in nanomedicine, such as how PEGylation reduces cellular interaction and how NP surface modifications impact therapeutic cargo delivery. In contrast to traditional cell inhibition assays that provide data at a single endpoint, live cell imaging offers continuous, quantitative assessment of morphological changes. This enables rapid screening of NP libraries based on the kinetics of drug response. While these experiments employed cytotoxic cargo, this morphology-based method is equally well-suited to non-toxic cargos that lead to changes in cell shape, size, or intracellular organization, such as differentiating agents. For example, we used this approach to observe the activation of dendritic cells treated with free and encapsulated poly-IC, which was clearly evidenced by morphological changes (Figure S6). In addition to this representative experiment shown here, HT can non-invasively assess cellular morphology, mechanics, and biochemical composition in any cell type, making it valuable for studying stem cell differentiation, neurological diseases, infectious disease pathology, and drug-induced cellular changes. There is an additional T cell Analysis Assay available with the EVE software. T cell analysis assays can be adapted to investigate immune responses in autoimmune disorders, transplant rejection, vaccine development, and chronic infections by characterizing T cell activation, cytokine production, and functional states. Together, these technologies enable comprehensive immune-cell interaction studies across diverse biological contexts, from understanding pathogen-host dynamics to optimizing regenerative medicine approaches.

### Kinetics of cargo delivery from solid lipid nanoparticles

**Figure 6.**
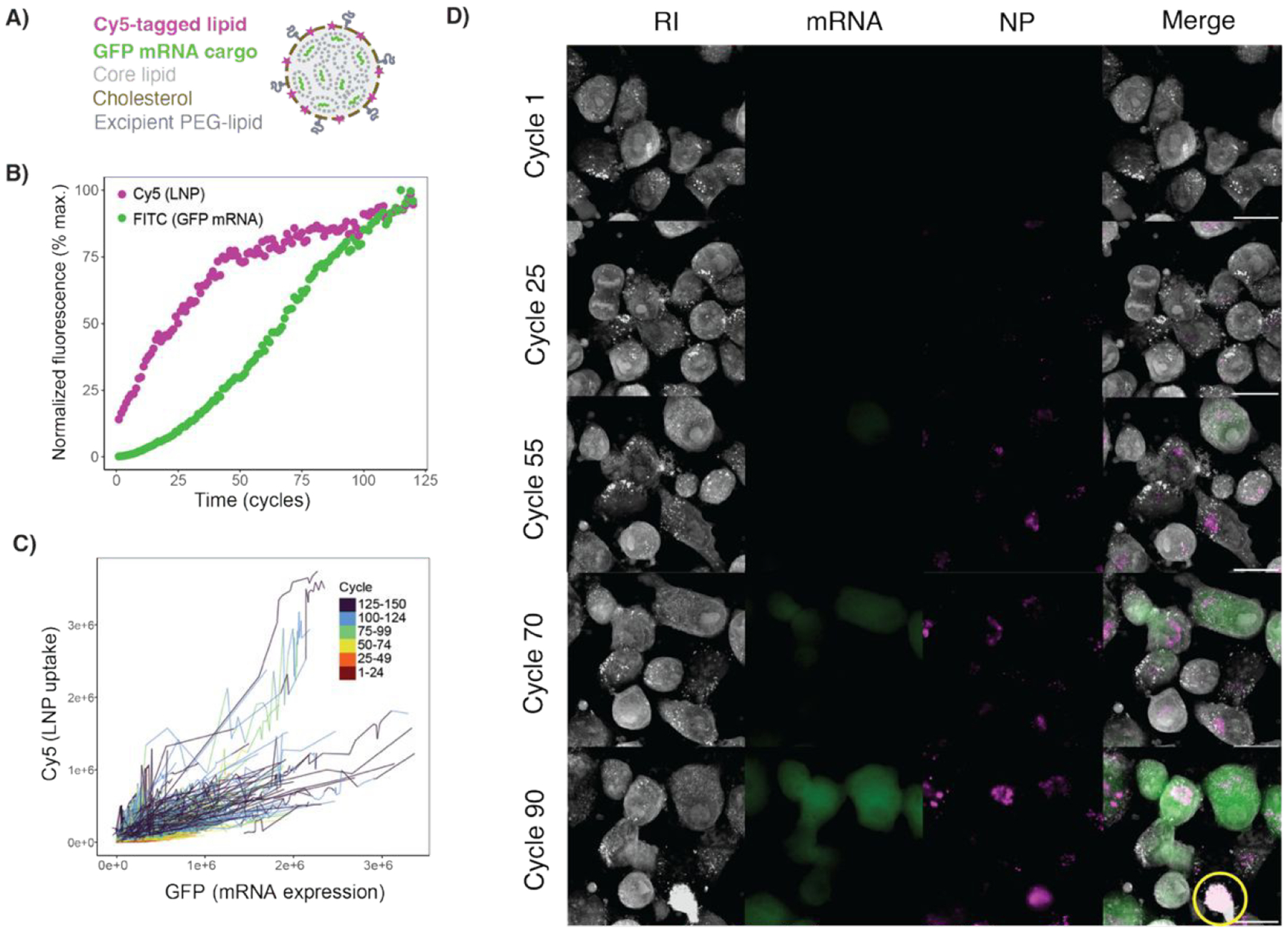
A) Schematic of the LNP formulation used with Cy5 pseudo-colored in magenta and GFP mRNA in green. B) Normalized fluorescence signal per-cell for the LNP and GFP mRNA cargo. C) Raw data for Cy5 (LNP) and FITC (GFP mRNA) signal for each cell in the FOV plotted per cycle. D) Representative images of an ROI at various cycles up to the point of phototoxic cell death (and signal saturation). Yellow circle indicates a cell that has been photobleached. Movies of the ROI shown in the figure and the whole FOV (8 fps). All scale bars are 20 μm.

Finally, we sought to investigate the kinetics of NP uptake and cargo delivery simultaneously using LNPs encapsulating nucleic acids. This NP-cargo combination has emerged as a transformative technology for vaccine delivery—with implications across many other diseases—and represents a powerful tool for basic cell biology research. We synthesized LNPs labeled with a Cy5-conjugated phospholipid and encapsulated messenger RNA (mRNA) encoding green fluorescent protein (GFP) and employed HT imaging with intermittent dual channel fluorescence (Figure 6A). To compare the kinetics of LNP update and cargo delivery, we monitored fluorescence intensity per cell at 8-minute intervals over 20 hours. This interval was chosen to maximize acquisitions, which mitigates phototoxicity (Figure S1). Morphologically, Cy5-LNP appeared as punctate intracellular signals that increased in intensity linearly over time, beginning almost immediately after dosing. In contrast, the GFP signal was diffuse and observed starting around cycle 25, or 3 hours after dosing (Figure 6B).

To further investigate the kinetics of LNP uptake and GFP expression, we utilized automated image analysis to perform cell segmentation and fluorescence quantification at both the population (Figure 6B) and individual cell levels (Figure 6C). At a population level, our data highlights the potential for simultaneously quantifying two key metrics of LNP drug delivery: uptake and mRNA translation. We observe that the Cy5 signal increases rapidly between Cycle 1–50, then plateaus. In contrast, the expression of the GFP mRNA cargo increased over time in a sigmoidal pattern. At the individual cell level, we found a positive correlation between Cy5 and GFP signals, with increasing variability noted over time. This variability may be attributable to heterogeneous LNP delivery and/or signal saturation that began around 30 hours after dosing (Figure 6D). The automated analysis allows us to normalize this variable signal and enhances the ability to observe discernible trends in LNP and cargo delivery at the population level.

The use of high-resolution, live cell imaging to parse the kinetics of LNP uptake and nucleic acid delivery in vitro has promising applications for mechanistic studies, such as investigating endosomal escape and intracellular cargo processing. Understanding these mechanisms within different biologic contexts may expand the utility of nucleic acid therapeutics. As a proof-of-concept, we used live cell imaging to validate previous work showing that expression of the solute carrier SLC46A3 negatively correlates with LNP uptake ^[13]^. By comparing LNP uptake cells genetically engineered to overexpress SLC46A3 and controls, we demonstrated that dynamic fluorescent holotomography can assess discrete differences in uptake kinetics. We confirmed these trends with flow cytometry, but its limitation to static time points further highlights the utility of live cell imaging as an orthogonal method for studying dynamic LNP delivery (Figure S4). It is important to note that flow cytometry provides a static snapshot of population-level cell characterization, which we show in Figure S4a at 20 hours, whereas in Figure S4b, this corresponds to the values at Cycle 100. With the use of fluorescent HT, we can capture up to 150 dynamic snapshots of the cell characterization within an automated workflow. This is a major advantage over traditional techniques that either require manual preparation for multiple time points or provide a single time-point perspective, reducing available kinetic information.

## Conclusions and Future Directions

Visualization of cellular interactions with NPs is critical for optimizing and designing highly efficient therapeutic nanocarriers. However, current high-throughput methods for characterizing NP-cell interactions are limited by reliance on chemical labels that can interfere with behavior, cell fixation that disrupts intracellular dynamics, and endpoint studies that capture static moments rather than continuous events.

Holotomography is a powerful label-free imaging technique that leverages the specific refractive indices of organelles to assess cell morphology, division, differentiation, and death. When paired with intermittent epifluorescence, it allows us to specifically track NP trafficking and cargo delivery without the need for organelle dyes or membrane stains. In this work, we present a method to monitor live cells and NP delivery in real time for up to 30 hours. By assessing a library of different NPs across adherent cell lines, we demonstrate the wide range of applications for the technique. Specifically, we visualize the cellular response to therapeutic NPs and track nucleic acid delivery, both of which are highly relevant applications in the field of nanotherapeutics.

While dynamic HT offers significant benefits for NP imaging, is also has certain limitations. First, for the instrument used in these studies, fluorescence imaging is confined to a single 2D imaging plane, unlike the 3D information captured by HT. Because this plane is set prior to acquisition, dynamic motion in the z-plane can be lost if a cell moves out of focus. Dead cells detaching from the surface can obscure the field of view, which can limit the duration of cell toxicity studies. In comparison with confocal fluorescence microscopy, which would reduce the out-of-focus signal from dead cells, we propose that the temporal resolution achievable with 2D fluorescence and 3D HT compensates for the information. Second, HT is best suited for adherent cells, and certain cell lines will require treated surfaces for enhanced attachment. As with other microscopy methods, plating conditions must first be optimized for each cell line based on size, seeding density, and proliferation rate. Finally, users must optimize several parameters for each experiment, including the NP-dye concentration and specific acquisition parameters. Additionally, it remains a limitation in the field to use fluorescent tags to observe nanoparticle trafficking; therefore, providing promising future development of high refractive index lipid NP formulations with the potential to be imaged solely using HT. Future studies will benefit from improvements in axial resolution or the ability to capture fluorescence at multiple imaging planes, which could mitigate some of these challenges. A potential suggestion for this advancement is to optimize the illumination for short-pulsed laser excitation to potentially reduce quenching or phototoxicity.

In conclusion, we present a new application of fluorescent holotomographic live-cell imaging to study NP uptake and delivery. This technique leverages the refractive index properties of cells and low-power illumination to capture dynamic, label-free cell behavior. By combining with intermittent fluorescence, we acquire qualitative and quantitative data to study NP localization and delivery kinetics. We also apply a robust automated imaging analysis software to robustly calculate metrics at an individual and population level, including cell count, area, subcellular contents, viability, apoptosis, and tracking of multiple fluorescence signals. Ultimately, this work addresses the critical need to preserve intrinsic cell dynamics over a prolonged period using live cell imaging, with significant implications for NP characterization and optimization.

This technology has promising future applications. The rich data generated could be used to create informative open-source datasets with diverse NP formulations and cell types. Such data could serve to train future models for cell-type detection or evaluation of NP uptake based on morphological changes. This technology is also well-suited for studying co-culture cell dynamics, an important and understudied aspect of NP design for cancer or immunological applications. The ability to make detailed temporal observations offers significant value for both for therapeutic and mechanistic studies.

## Supplementary Material

Supplementary material

## Figures and Tables

**Table 1.** Benefits and potential limitations for use of fluorescent holotomography.

Optimal for:	Limitations:
Label-free, long-term, live cell imaging unattainable with phase or confocal Assessing adherent cell morphology, division, or differentiation Longitudinal response to therapeutics Co-culture cell-cell interactions Fluorescent NP tracking Delivery or expression kinetics Providing quantitative information independent of phase delay	Single plane for fluorescence 200 nm lateral resolution Requires precise seeding density optimization, dependent on the cell line Adherent cells in a monolayer only Potential phototoxicity with high laser power or prolonged exposure

## Data Availability

Data for this article, including raw, high-resolution TIFF files, are available on Dryad at Dataset DOI: 10.5061/dryad.zkh1893qf. Select data supporting this article have been included as part of the Supplementary Information. Nanolive SA, Switzerland, analysis software (EVE) was used for the data analysis.

## New Concepts Statement

In this study, we demonstrate a novel application of holotomography (HT) combined with fluorescence microscopy, enabling quantitative, long-term observation of nanoparticle-cell interactions with reduced phototoxicity or artifacts inherent to conventional microscopy approaches. HT resolves subcellular features like lipid droplets, mitochondria, plasma membrane, nuclei and nucleoli, based on intrinsic refractive index, allowing continuous tracking of cell- and population-level dynamics. Incorporating intermittent fluorescence microscopy, we expand this technique to observe nanoparticle trafficking and cargo delivery in cancer cells for over 30 hours with minimal photodamage. This multimodal method advances nanoscience by bridging subcellular resolution with nanoparticle uptake kinetics. Leveraging quantitative analysis pipelines, cells can be categorized based on morphology and fluorescence signal, capturing cargo delivery and pharmacodynamic metrics simultaneously. Combining a low-power laser for HT and controlled intermittent fluorescence acquisition reduces photobleaching while maintaining spatial and temporal resolution sufficient to resolve dynamic cellular and subcellular processes. This brings critical new insights to nanotherapeutic design by facilitating mechanistic studies of uptake and cargo delivery across heterogeneous cells–information that is typically inaccessible with endpoint assays, short-term imaging, or imaging fixed cells. This HT workflow transforms nanoparticle characterization from static snapshots to a dynamic assessment of cell behavior and nanoparticle interactions.

## Acronyms:

RI: refractive index
BF: brightfield
HT: holotomography
NP: nanoparticle
LNP: lipid NP
PS: polystyrene
PEG: poly(ethylene glycol)
PLE: poly-L-arginine
PLD: poly-L-aspartate
PLE: poly-L-glutamate
HA: hyaluronic acid
poly-IC: polyinosinic-polycytidylic acid
LOX: LOX-IMVI
