# Supplementary material for: Subcellular nanoparticle trafficking investigated with label-free, live cell imaging
Source: Nanoscale Horiz. Author manuscript; Available in PMC 2026 Feb 27. (PMC12914502; doi:10.1039/d5nh00749f)
Supplement: Supplementary material [file NIHMS2149521-supplement-Supplementary_material.pdf]

Supplementary Information (SI) for Nanoscale Horizons.
This journal is © The Royal Society of Chemistry 2026

Supplemental figures:

Table S1. Characterization of NPs used in this study

Formulation	Composition	Z-average diameter	PDI	Zeta potential
Bare liposome	31:31:31:7 DSPC:DSPG:Cholesterol:Cy5-DSPE	94.77 ± 2.38	0.21 ± 0.08	-69.67 ± 6.3
Polystyrene (carboxylated)	Obtained commercially: carboxylate-modified latex, loaded with commercial 505/515 dye	82.43 ± 3.9	0.25 ± 0.01	-48.6 ± 2.6
LNP	50:38.5:10:1.5 ALC-0315:Cholesterol: DOPE:DMG-PEG2k	103.9 ± 1.5	0.158 ± 0.008	22.6 ± 1.5
CPX-351	Obtained commercially: 7:2:1 molar ratio DSPC:DSPG:cholesterol	129.0 ± 2.1	0.06 ± 0.03	-47.49 ± 0.1

Table S2. Technical Specifications

Illumination (HT):	Low power laser (λ = 520 nm, Class I)<br>Sample exposure = 0.2 mW/mm^2<br>Speed: high (<100 μs)
Resolution:	Holotomography (3D): lateral (xy) = 200 nm, axial (z) = 400 nm<br>Fluorescence (2D): xy = 400 nm
Objective:	60x magnification, air objective, NA 0.8
Field-of-view (1x1):	Holotomography: 90 x 90 x 30 $μm^3$<br>Fluorescence: 90 x 90 $μm^2$<br>35 mm dish: up to 5x5 grid scan<br>96 well plate: up to 3x3 grid scan
Epifluorescence:	Filters for FITC (488 nm) | Cy5 (647 nm) | DAPI (390 nm)
Modality:	Automated 3D holotomography (96 slices) + epifluorescence (1) + time-lapse.<br>The minimum number of slices acquired is 96 with a step size of 0.40 μm, providing a reconstructed 3D volume of approximately 30 μm.
Settings:	Cy5: 10-15% power, 80-100 ms exposure, 30-50 gain<br>FITC: 5-12% power, 50-150 ms exposure, 15-40 gain<br>Images analyzed with Cytotoxicity and Lipid Droplet Assays.<br>3x3 and 4x4 grid scans acquired with RI every 5-12 minutes and fluorescence every

	7-12 minutes (when used).

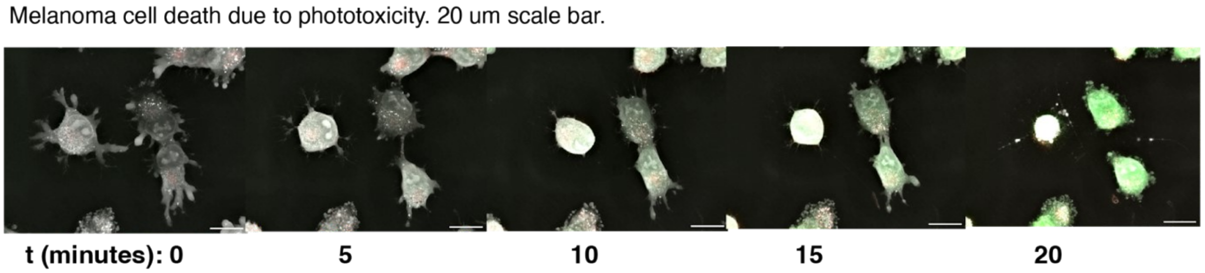

Figure S1. Phototoxicity-induced cell death via apoptosis, which is observed when the cell becomes circular and uniformly bright. Cells detach after undergoing apoptosis. LOX cells were imaged at 488 and 647 nm every 5 minutes (~3.3 mHz) with both laser powers set to 15%. All scale bars are 20 μm. Movie (8 fps).

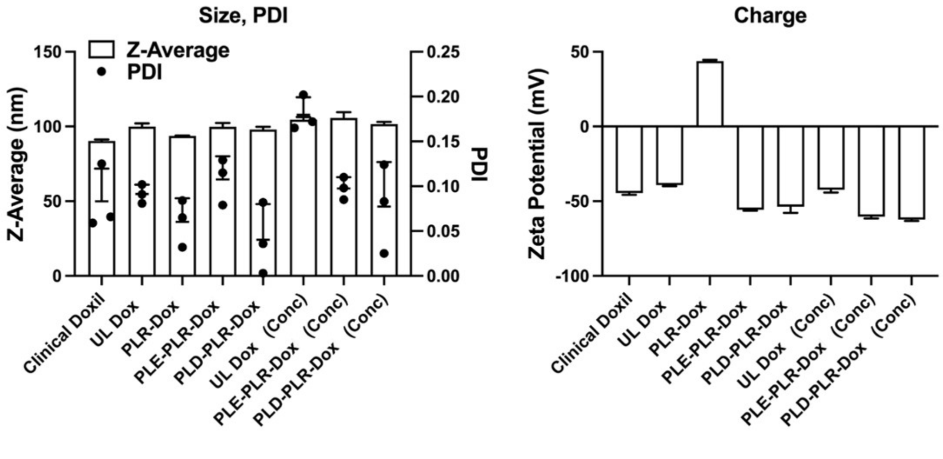

	[Dox]	[Lipid]	[Dox]/[Lipid]
UL Doxil:	4.38 mg/mL	31.395 mg/mL	0.140
PLE-PLR-Doxil (Conc):	3.95 mg/mL	38.483 mg/mL	0.103
PLD-PLR-Doxil (Conc):	2.51 mg/mL	12.326 mg/mL	0.204

Figure S2. Doxorubicin-loaded NP (abbreviated “Dox”) characterization of size, zeta potential, effective concentration after loading, and ratio of drug to lipid.

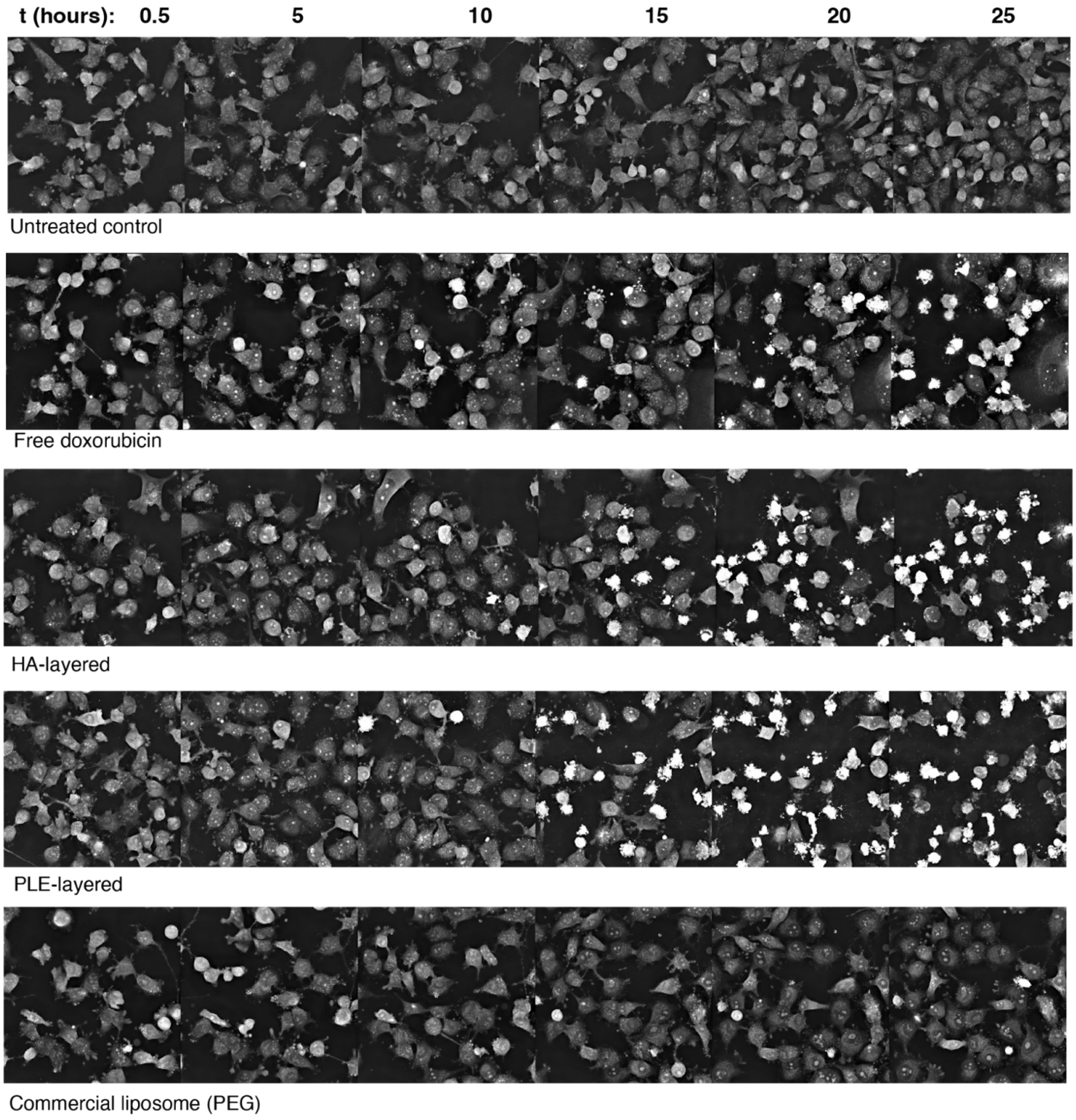

Figure S3. Representative time-lapse images for each condition in the layer-by-layer doxorubicin-loaded particle experiment from Figure 4. Cells that undergo apoptosis appear highly refractive and bright.

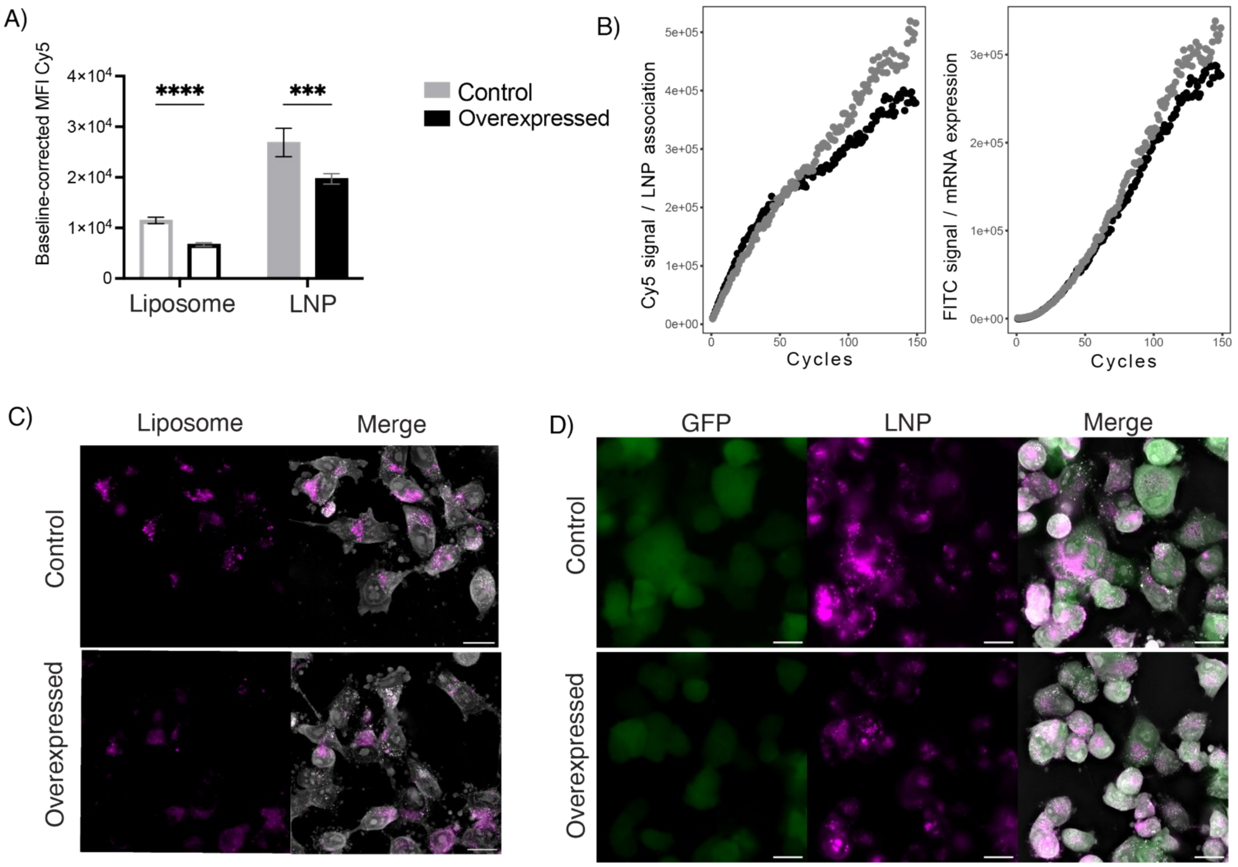

Figure S4. A) Bare liposome and LNP association measured by flow cytometry 20 hours after dosing LOX cells. Evaluated by unpaired t-test (***p<0.01 and ****p<0.001). B) LNP association measured by Cy5 and FITC signal with fluorescent HT imaging over 20 hours (150 cycles at ~2 mHz). At roughly Cycle 100 is the static timepoint equivalent to the timepoint chosen for flow cytometry. C) Representative images of bare liposome uptake at cycle 100 in unmodified LOX cells compared to LOX cells overexpressing (OE) SLC46A3. D) Representative images of LNP uptake at cycle 100 in unmodified cells compared SLC46A3 OE cells. All scale bars are 20 μm.

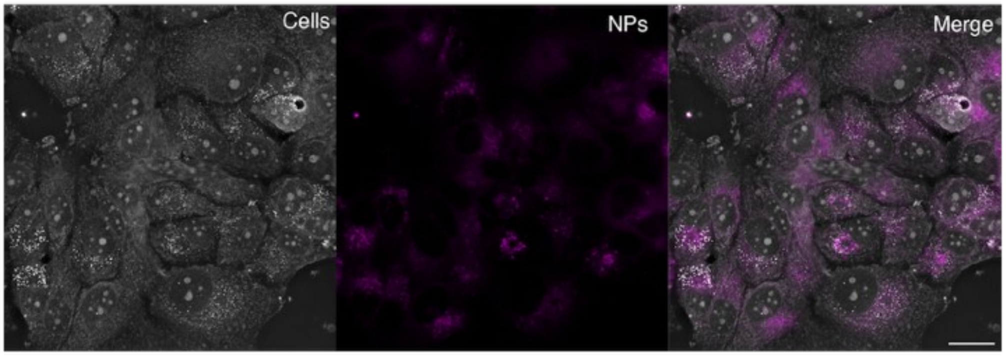

Figure S5. Representative image of T47D cancer cells after taking up bare liposomes. 20 μm scale bar. Movie (6 fps, 20 μm). Additional observation of endocytosis captured in the Movie (6 fps, 20 μm).

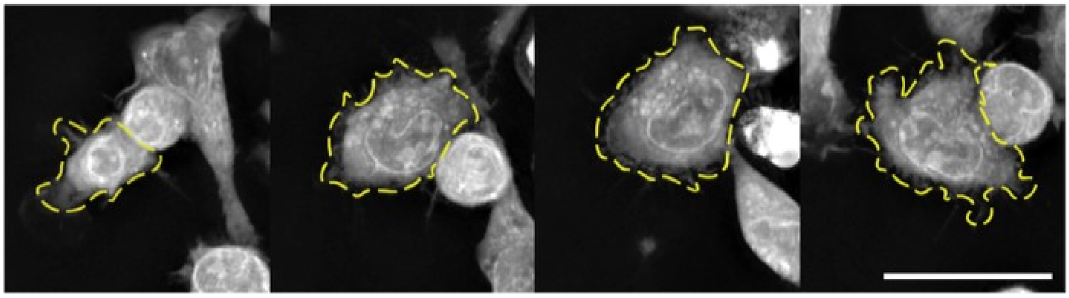

Figure S6. Representative images of a dendritic cell being activated by treatment with poly-IC. The cell is traced in yellow to show how the morphology of a dendritic cell changes upon activation by an agonist.

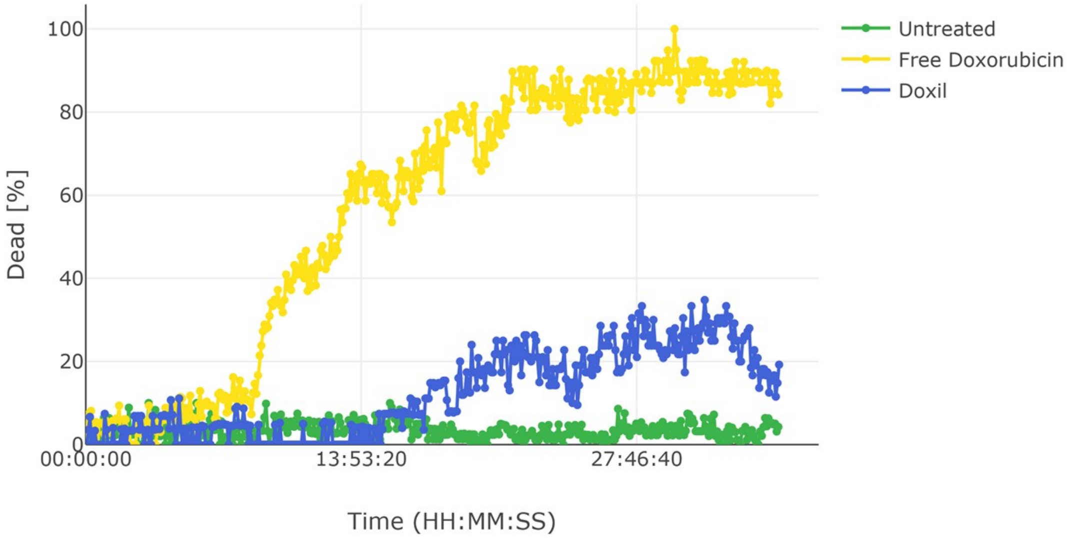

Figure S7. Sample representation of raw quantitative data for the doxorubicin liposome experiments. This example used the cell metrics for percent dead over time processed with auto-segmentation for dead cell detection.

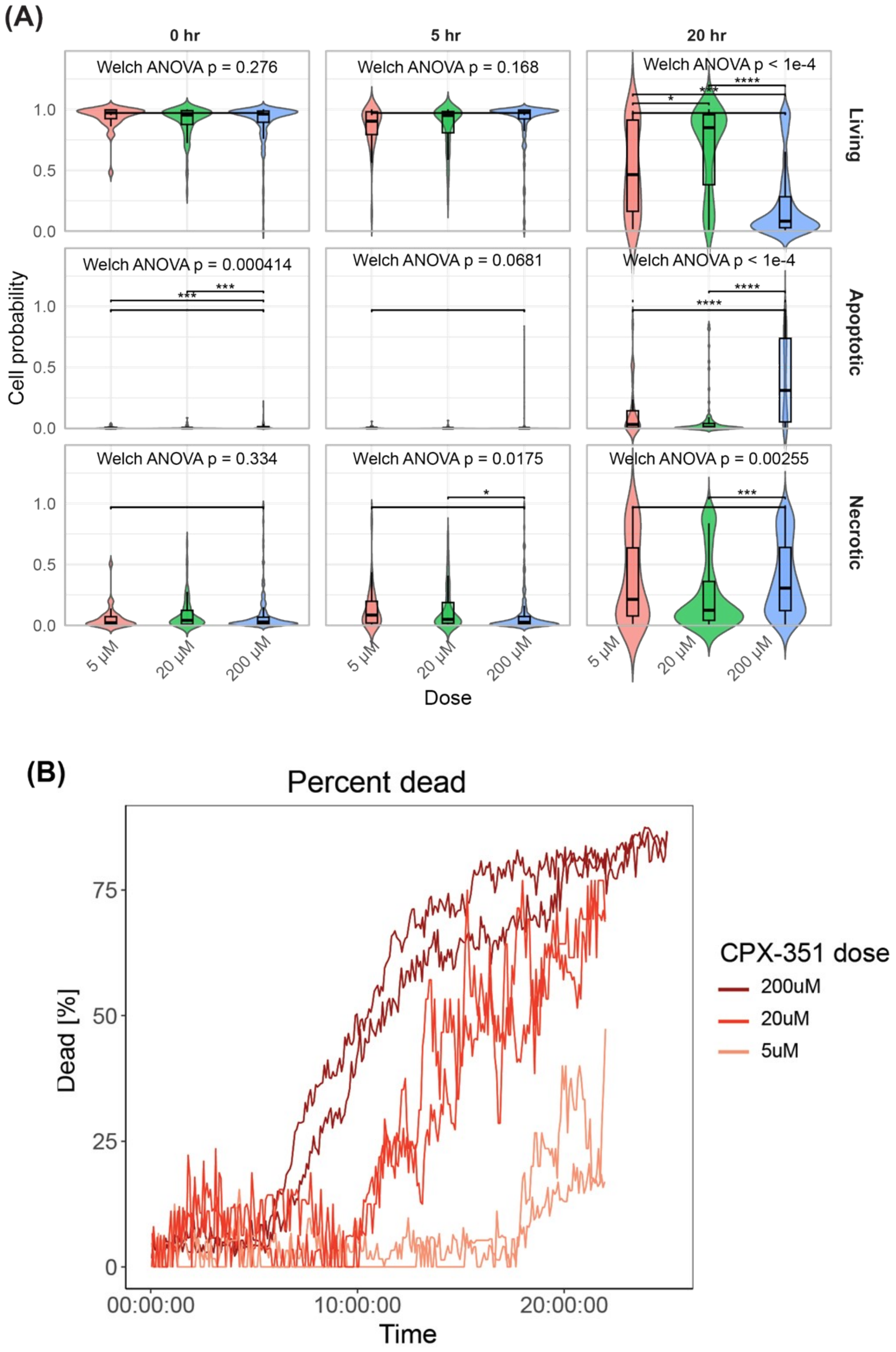

Figure S8. (A) At each time point, cell-state probabilities were compared across doses using Welch's one-way ANOVA to accommodate unequal variances, with post-hoc pairwise differences assessed by the Games–Howell test. Tests were two-sided, conducted independently per time point and cell state, and Games–Howell p-values were used for multiplicity control within each time point. (B) Sensitivity to the variability of the three doses used (5, 25, and 250 μM) was assessed in duplicate, demonstrating consistent sensitivity within the Cytotoxicity Assay analysis.

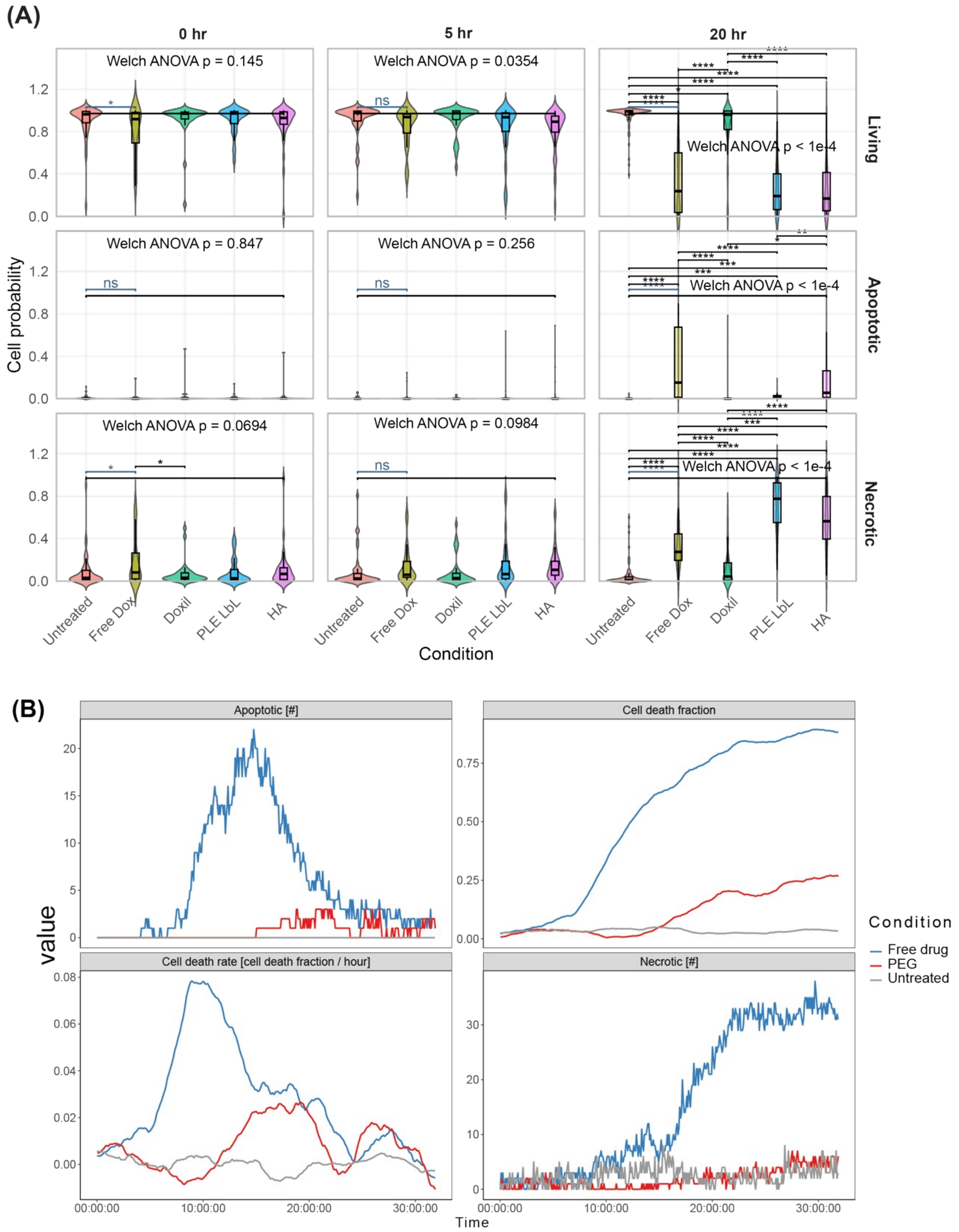

Figure S9. (A) Cell fate probabilities were compared across treatment groups at each time point using Welch's one-way ANOVA to accommodate unequal variances. Post-hoc pairwise differences were assessed using the Games–Howell test, and a pre-specified comparison between Untreated and Free Drug was evaluated using a Welch two-sample t-test; all tests were two-sided.(B) Population-level assessment of free doxorubicin compared to an unlayered PEG-only liposome and the untreated control group to establish the quantifiable level of variability between control groups.

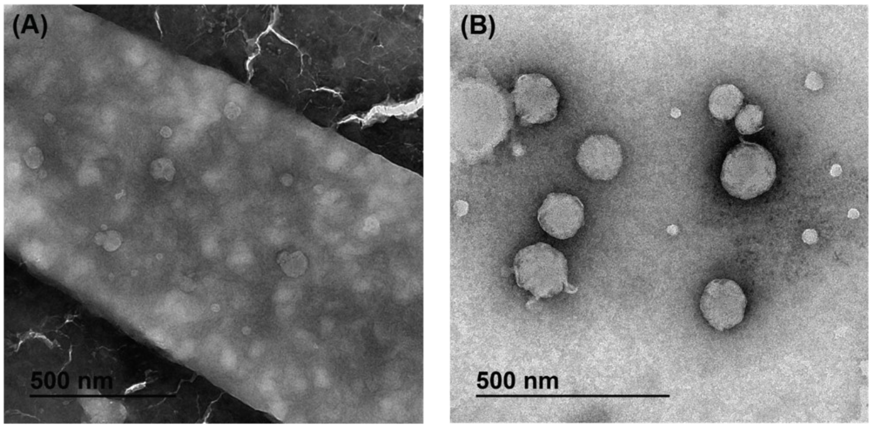

Figure S10. Representative transmission electron microscopy images of the (A) HA and (B) PLE layered nanoparticle formulations. Method: 10 uL of LNPs and buffer containing solution was dropped on a 200 mesh copper grid coated with a continuous carbon film. 10uL of negative staining solution, phosphotungstic acid (1% aqueous solution), was dropped on the TEM grid and immediately removed by KimWipes. Then, 10uL of the stain was applied to the grid; after 30 seconds, the excess stain was removed by touching the edge with KimWipes. The grid was dried at RT, then mounted on a JEOL single tilt holder equipped in the TEM column. The specimens were cooled down by liquid nitrogen; imaging on an JEOL 2100 FEG microscope was done using a minimum dose method that was essential to avoid sample damage under the electron beam. The microscope was operated at 200 kV and with a magnification in the range of 10,000-60,000 for assessing particle size and distribution. All images were recorded on a Gatan 2kx2k UltraScan CCD camera.

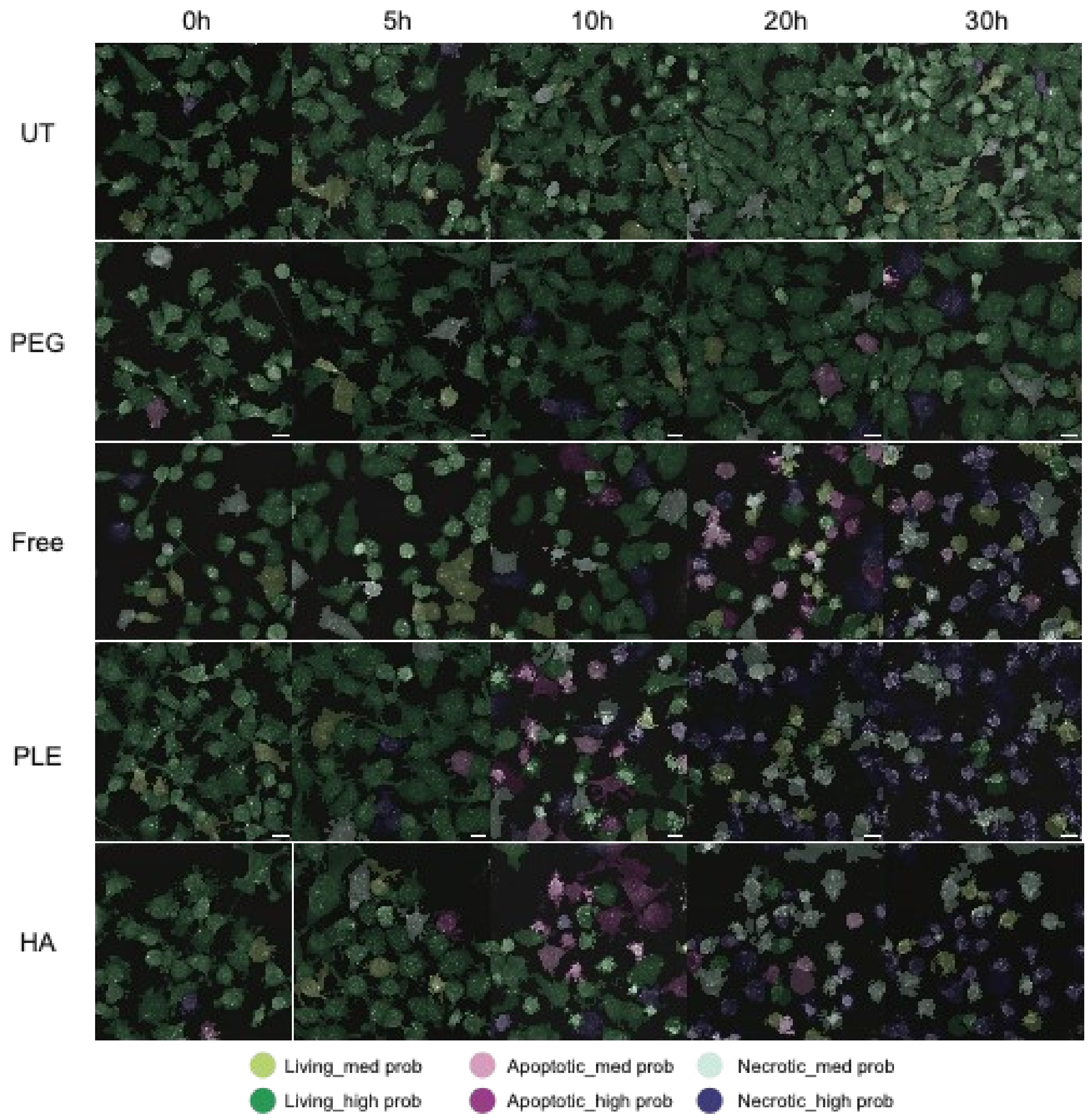

Figure S11. Representative time-lapse images of cell death over time following treatment with doxorubicin-loaded nanoparticles in the segmentation and masking steps of the automated analysis. Living cells are masked in green, apoptotic cells are masked in purple, and necrotic cells are masked in blue. The masks are applied using the EVE Live Cytotoxicity Assay.
